# Molecular Basis for Drug Resistance in HIV-1 Protease

**DOI:** 10.3390/v2112509

**Published:** 2010-11-12

**Authors:** Akbar Ali, Rajintha M. Bandaranayake, Yufeng Cai, Nancy M. King, Madhavi Kolli, Seema Mittal, Jennifer F. Murzycki, Madhavi N.L. Nalam, Ellen A. Nalivaika, Ayşegül Özen, Moses M. Prabu-Jeyabalan, Kelly Thayer, Celia A. Schiffer

**Affiliations:** 1 Department of Biochemistry and Molecular Pharmacology, University of Massachusetts Medical School, Worcester, Massachusetts 01605, USA; E-Mails: Akbar.Ali@umassmed.edu (A.A.); Rajintha.Bandaranayake@umassmed.edu (R.M.B.); Yufeng.Cai@umassmed.edu (Y.C.); Nancy.King@umassmed.edu (N.M.K.); Madhavi.Kolli@umassmed.edu (M.K.); Seema.Mittal@umassmed.edu (S.M.), Madhavi.Nalam@umassmed.edu (M.N.L.N.); Ellen.Nalivaika@umassmed.edu (E.A.N.); Aysegul.Ozen@umassmed.edu (A.Ö.); Kelly.Thayer@umassmed.edu (K.T.); 2 Department of Pediatrics, University of Rochester, Rochester, NY 14627, USA; E-Mail: jennifer.murzycki@gmail.com; 3 Division of Basic Sciences, The Commonwealth Medical College, 150 N. Washington Avenue, Scranton, PA 18503, USA; E-Mail: mprabu@tcmedc.org

**Keywords:** drug resistance, HIV-1 protease, protease inhibitors, substrate envelope, structure based drug design

## Abstract

HIV-1 protease is one of the major antiviral targets in the treatment of patients infected with HIV-1. The nine FDA approved HIV-1 protease inhibitors were developed with extensive use of structure-based drug design, thus the atomic details of how the inhibitors bind are well characterized. From this structural understanding the molecular basis for drug resistance in HIV-1 protease can be elucidated. Selected mutations in response to therapy and diversity between clades in HIV-1 protease have altered the shape of the active site, potentially altered the dynamics and even altered the sequence of the cleavage sites in the Gag polyprotein. All of these interdependent changes act in synergy to confer drug resistance while simultaneously maintaining the fitness of the virus. New strategies, such as incorporation of the substrate envelope constraint to design robust inhibitors that incorporate details of HIV-1 protease’s function and decrease the probability of drug resistance, are necessary to continue to effectively target this key protein in HIV-1 life cycle.

## 1. Introduction

According to the recent reports published by UNAIDS, there are about 33.4 million people living with HIV-AIDS around the globe [1]. Currently, there is no permanent cure or vaccine for AIDS but there are about 25 drugs that belong to seven classes targeting different stages in the life cycle of HIV [2]. Although the quality and life expectancy of HIV infected patients has improved since the introduction of antiviral treatment, low drug adherence, toxicity, and high pill burden, coupled with the error prone mechanism of HIV reverse transcriptase, have led to the emergence of drug resistance in HIV infected patients (for recent reviews see [2–5]).

**Figure 1.**
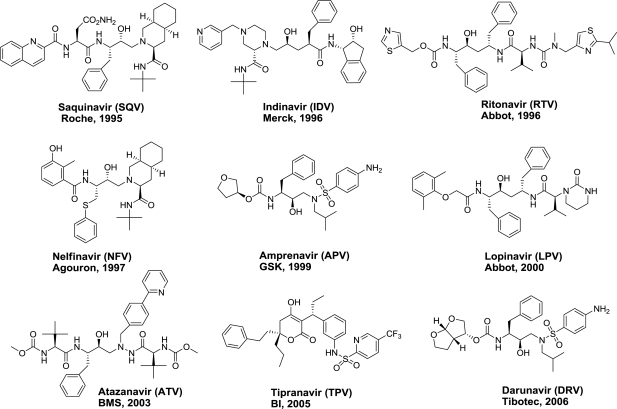
FDA-approved HIV-1 protease inhibitors.

Protease inhibitors (PIs) are one class of drugs that target an essential viral enzyme HIV-1 protease. Because of its critical role in the processing of Gag and Gag-Pro-Pol polyproteins into individual proteins necessary for viral maturation [6], protease is one of the major therapeutic targets for developing antiviral drugs against HIV-AIDS. In the last two decades, drug discovery efforts aided by structure-based design have led to the development of nine FDA-approved protease inhibitors (PIs) (Figure 1): Saquinavir (SQV) [7], Indinavir (IDV) [8], Ritonavir (RTV) [9], Nelfinavir (NFV) [10], Amprenavir (APV) [11], Lopinavir (LPV) [12], Atazanavir (ATV) [13], Tipranavir (TPV) [14], and Darunavir (DRV) [15–17]. These inhibitors represent the most potent anti-AIDS drugs reported to date and are essential components of the highly active antiretroviral therapy (HAART) [18,19]. HAART is credited with significantly reducing AIDS-related mortality [20,21] and is currently implemented throughout the world as the standard of care for HIV-AIDS treatment.

Drug resistance to PIs has become a major issue with the failure of HAART. Moreover, newly infected patients are infected with resistant viruses which are an added challenge in the treatment of HIV infections. Various strategies have been used to develop new antiviral therapies against drug-resistant HIV, including increasing the plasma levels of existing PIs by using a boosting agent [22] and developing new PIs using structure-based drug design [4,23–25]. Among different approaches, one design strategy maximizes the number of hydrogen bonds with the protease backbone and led to the development of highly potent PIs active against drug-resistant HIV [25,26]. PIs with improved resistance profiles were also developed using a solvent anchoring approach [27], and utilizing a new lysine sulfonamide-based molecular core [28]. Another design strategy incorporates substrate envelope constraints into structure-based design and led to the discovery of novel highly potent PIs that are less susceptible to drug-resistance [29]. The principles underlying these various strategies are not necessarily mutually exclusive and all achieved the design of highly potent inhibitors against drug-resistant HIV.

## 2. FDA-approved HIV-1 Protease Inhibitors

**Figure 2.**
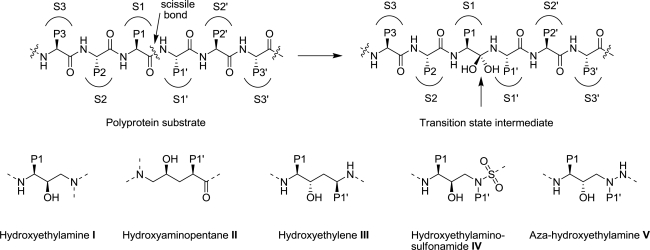
The scissile bond in polyprotein substrate is hydrolyzed by protease through the transition state intermediate (substrate amino acid residues are marked as...P3, P2, P1, P1′, P2′, P3′…and the corresponding enzyme binding sites as…S3, S2, S1, S1′, S2′, S3′…). Transition state mimics I–V used in the design of currently approved drugs.

All currently approved HIV-1 PIs are competitive active site inhibitors that bind in the active site of the protease and, except for TPV, all are peptidomimetics. These PIs were rationally designed based on the transition state mimetic concept and contain various non-cleavable dipeptide isosteres as core scaffolds to mimic the transition state of the polyprotein substrates of HIV-1 protease (Figure 2). A key common feature of these inhibitors is the presence of a secondary hydroxyl group, a surrogate for the P1 carbonyl moiety of substrates, which makes critical interactions with the catalytic Asp25/25′ residues of the protease and is required for tight inhibitor binding with the protease. Another common feature in the complexes between peptidomimetic inhibitors and HIV-1 protease is a conserved water molecule that mediates contacts between the P2/P1′ carbonyl oxygen atoms of the inhibitors and the amide groups of Ile50/Ile50′ of the enzyme. The development and clinical introduction of HIV-1 PIs is regarded as a major success of structure-based rational drug design [30].

Development of the first generation PIs was greatly facilitated by the knowledge of inhibitors of other aspartic proteases such as renin, and early availability of numerous crystal structures of both unliganded enzyme and enzyme-ligand complexes [30–32]. Initial designs of inhibitors were based on pepstatin, a natural transition state mimic, and sequence homology of substrate cleavage sites at the Gag and Gag-Pro-Pol polyprotein containing a non-cleavable reduced amide dipeptide isostere [33]. The crystal structures of these early inhibitor-protease complexes provided a wealth of information on the inhibitor-enzyme interactions in the protease active site and led to the optimization of various lead inhibitors.

SQV, discovered by Roche [7], was the first HIV-1 PI approved by the FDA in December 1995 for the treatment of HIV-AIDS. The initial pentapeptide lead was based on the HIV-1 pol substrate sequence containing the unusual Phe-Pro amide bond at the cleavage site. Lead optimization, including replacement of the P1-P1′ amide bond with non-cleavable hydroxyethylamine (Figure 2-I) based dipeptide isostere, replacement of the P1′ proline with a bicyclic decahydroisoquinoline, and introduction of a quinoline moiety at P3 led to the discovery of SQV. Although SQV is a very potent (*K*i = 0.12 nM) and selective inhibitor of HIV-1 protease, SQV has very poor bioavailability and is quickly degraded *in vivo* by cytochrome P450 (CYP-450).

SQV was soon followed by two structurally distinct PIs, IDV [8] and RTV [9]. IDV, developed by Merck, was also optimized from an initial peptide lead in which the P1-P1′ fragment was replaced with a novel Phe-Gly hydroxyaminopentane dipeptide isostere (Figure 2-II). The other key structural features of IDV are the aminohydroxyindane moiety at P2′ position and a P1-P2 pyridylmethylpiperazine moiety. IDV has protease inhibitory potency of 0.6 nM, antiviral potency of 25–100 nM, and has excellent oral bioavailability.

In the discovery of RTV, the Abbott team sought to exploit the C2 symmetry of the HIV-1 protease and initially designed inhibitors by incorporating a C2 symmetric dihydroxy Phe-Phe isostere core. During the lead optimization process, they discovered that the second hydroxyl group in the core isostere could be removed without affecting the potency leading to the development of a pseudo-symmetric all carbon Phe-Phe hydroxyethylene isostere core (Figure 2-III). RTV potently inhibits HIV-1 protease (*K*i = 0.022 nM) and has moderate antiviral potency (EC_50_ = 60 nM). Due to its numerous side effects RTV is no longer used as a PI on its own. However, RTV is a potent inhibitor of CYP-450 3A4 isoform [34], and, because of this side activity, low dose RTV is currently used as a boosting agent in HAART therapy with other PIs.

NFV [10] was developed by truncating the N-terminal moiety in SQV and replacing the P2 asparagine with 3-hydroxy-2-methylbenzamide fragment. These changes in combination with a novel P1 moiety in the hydroxyethylamine isostere led to NFV (*K*i = 2 nM) with significantly reduced molecular weight and improvement in bioavailability, though NFV is less potent than SQV. Efforts by Vertex aimed at reducing the molecular weight and peptide character of PIs led to the discovery of APV [11], which incorporates a novel hydroxyethylamino-sulfonamide dipeptide isostere (Figure 2-IV). The 3-hydroxyltetrahydrofuran P2 moiety was designed to mimic the interactions of SQV’s asparagine side chain with the Asp29 residue. APV, also approved as a prodrug (fosamprenavir), is the smallest of the 9 currently approved PIs; APV has moderate potency (*K*i = 0.6 nM), good bioavailability and long half-life, allowing twice daily dosing in patients.

Based on the first generation PI RTV, Abbott developed a highly potent second generation inhibitor, LPV [12], which is also active against RTV-resistant protease variants. Significant efforts directed at replacing the bulky (2-isopropylthiazolyl)methyl P3′ moiety with smaller groups led to the discovery of cyclic urea as a high affinity P3′ moiety. The P2 thiazolylmethyl moiety was also replaced with a more lipophilic 2-(2,6-dimethylphenoxy)acetamide resulting in an exceedingly potent PI with 10-fold better potency than RTV. Although LPV has poor bioavailability and pharmacokinetic profile, its plasma levels could be significantly enhanced by adding low dose RTV [22]; a combination of LPV/RTV (Kaletra) is one of the most widely used PI therapies.

ATV [13], approved in 2003, incorporates a novel (hydroxyethyl)hydrazine or aza-hydroxyethylamine dipeptide isostere (Figure 2-V), an extended 4-(2-pyridinyl)phenylmethyl moiety and a methylcarbamate capped tert-leucine moiety at both P2/P3 and P2′/P3′ positions. Compared to the hydroxyethylene core of LPV, the P1–P2 aza-linkage eliminates one of the three chiral centers allowing easier large-scale synthesis. ATV has high antiviral potency and oral bioavailability, and is the only PI that allows once daily dosing.

TPV [14] is the only non-peptidomimetic PI developed from lead compounds 4-hydroxycoumarin and 4-hydroxy-2-pyranone, identified by high throughput screening. Unlike other PIs, TPV is not a transition state mimetic, and instead binds to the protease in a distinct fashion replacing the conserved flap water. The phenolic hydroxyl group of the central 4-hydroxy-2-pyranone moiety makes hydrogen bond interactions with the Asp25/25′ in the floor of the active site and the carbonyl group, unlike peptidomimetic inhibitors, makes direct hydrogen bond interactions with Ile50/50′ in the flap region of the protease. TPV potently inhibits multidrug-resistance protease variants and the replication of viruses that are resistant to most other PIs. TPV, due to its unique binding mode with the protease, a resistance profile different from other drugs, and a higher barrier to resistance requiring multiple mutations, is recommended for therapy with patients containing preexisting protease resistance.

DRV [15–17], the latest protease inhibitor approved by the FDA, incorporates the same hydroxyethylamino-sulfonamdie isostere present in APV. In fact, both compounds are very similar with the only difference being a condensed bis-tetrahydrofuranyl (bis-THF) moiety at P2 present in darunavir instead of a single tetrahydrofuranyl (THF) ring of APV. DRV was developed by both academic and industrial research efforts based on the crystal structures of HIV-1 protease bound to APV, SQV and its analogues bearing the bis-THF moiety at P2 position. These crystal structures revealed that the oxygen atoms of the THF/bis-THF moieties make extensive hydrogen bond interactions with the Asp29/Asp30 residues of the protease enzyme. The critical interactions of the bis-THF moiety in the S2 binding pocket of the protease enzyme are largely responsible for the exceptionally high inhibitory and antiviral potency of darunavir (*K*i = 15 pM; EC_50_ = 1–4 nM). DRV is the most potent antiviral protease inhibitor approved to date and is also highly effective against most of the multi-drug resistant HIV-1 variants.

The enzyme inhibitory activities of all FDA approved HIV PIs against wild-type (WT) protease and three drug-resistant variants and their cellular antiviral potencies against wild-type HIV are provided in Table 1 for comparison. The first generation PIs, RTV, SQV, IDV, NFV, lose significant activity against drug-resistant protease variants, however, recently approved drugs TPV and DRV retain low picomolar (pM) inhibitory activities.

## 3. Interdependency of Drug Resistance

### 3.1. Substrate Envelope Hypotheses

**Figure 3.**
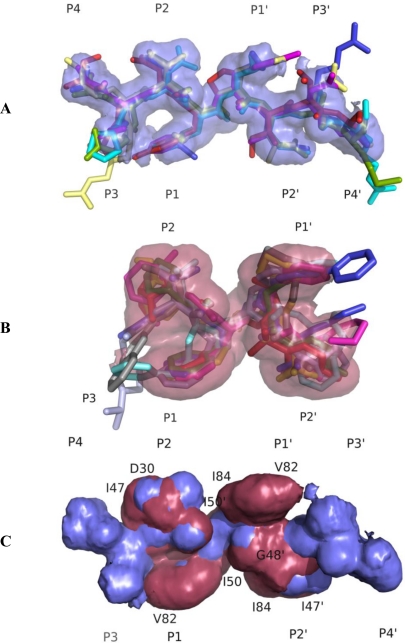
**(A)** Substrate envelope of HIV protease. PyMOL model generated from overlapping van der Waals volume of substrate peptides. Red: matrix capsid, green: capsid-p2, blue: p2-nucleocapsid, cyan: p1–p6, magenta: reverse transcriptase-ribonucleaseH, yellow: ribnucleaseH-integrase. **(B)** The inhibitor envelope in red, within the active site of HIV-1 protease, calculated from overlapping van der Waals volume of five or more of eight inhibitor complexes. **(C)** Superimposition of the substrate consensus volume (blue) with the inhibitor consensus volume (red). Residues that contact with the inhibitors where the inhibitors extend beyond the substrate volume and confer drug resistance when they mutate are labeled (Figures 3A–C, modified from King *et al*. [38]).

Within the Gag and Gag-Pro-Pol, HIV-1 protease cleavage sites are non-homologous and asymmetric, both in charge and size. These characteristics begged the question as to how a symmetric protease could recognize and cleave an asymmetric substrate. Structural studies have shown that the various cleavage site peptides adopt a conserved shape/volume, which was hypothesized as the basis for recognition of substrate sites by the HIV-1 protease [37]. This overlapping volume of the majority of the substrates within the active site of the protease defines the conserved shape or the “substrate envelope” (Figure 3A). The P1–P3 region of the substrates forms a toroid, which is thought to be important for specificity, whereas the numerous backbone-to-backbone interactions of the protease and the substrates facilitate binding [37]. The substrate envelope not only explains specificity of the substrates but also the development of resistance to various PIs and substrate co-evolution [38].

Crystallographic studies of the wild-type protease bound to inhibitor molecules have shown that most of the PIs occupy a similar volume (defined as the inhibitor envelope, Figure 3B) and contact similar residues within the active site of the protease. Drug resistance occurs where inhibitor atoms protrude beyond the substrate envelope and contact protease residues (Figure 3C) [38]. Thus, mutations at these sites would specifically impact inhibitor binding while substrate recognition and cleavage remains relatively unaffected. The fact that most of the sites of drug resistant mutations in the active site do not contact the substrates led to the development of the substrate envelope hypothesis: Inhibitors that fit well within the substrate envelope would be less susceptible to drug resistance, as a mutation that affects inhibitor binding would simultaneously impact the recognition and processing of the majority of the substrates [38]. Of the currently prescribed inhibitors the most efficacious is DRV and although not designed using the substrate envelope constraint, DRV fits well within this volume [39,40]. These studies also suggested that if the substrate atoms that protrude out of the substrate envelope contact the very same residues in the active site of the protease that mutate to prevent inhibitor binding, it could lead to impaired substrate recognition and cleavage resulting in the co-evolution of compensatory mutations within the protease cleavage sites [38].

### 3.2. Drug Resistance—A Change in Molecular Recognition at the Active Site

The development of drug resistance is a major factor for the failure of protease inhibitor therapy. The virus evolves to accumulate a multitude of mutations within the protease that prevent PIs from binding to the protease. More than half the residues within the protease mutate in different combinations and lead to drug resistance [41,42]. Drug resistance is a subtle change in the balance of recognition events: The protease is still able to recognize and process the natural substrate sites in the Gag and Gag-Pro-Pol polyprotein, while no longer being effectively inhibited by competitive drug molecules. This hints that as drug resistance emerges, the interactions of the protease with an inhibitor should significantly be altered to facilitate the reduced affinity of the protease to the inhibitors while the interactions with a natural substrate should be maintained as in the wild-type structures.

As the functional HIV-1 protease is a symmetric dimer, both monomers contribute to substrate binding. The active site region is primarily formed by residues 25–32, 47–53 and 80–84. Mutations occurring anywhere else in the protease are referred to as the non-active site mutations.

Under protease inhibitor therapy, a majority of initial mutations arise within the active site of the enzyme, directly affecting inhibitor binding and are the primary cause of resistance to PIs. Typical primary mutations include D30N, G48V, I50L/V, V82A/F/T, I84V and L90M [43]. Several primary PI resistance mutations have been described that are a signature of particular PIs. For example, patients failing NFV therapy develop the D30N protease mutation [44], while the I50V and I50L mutations are selected in patients failing APV/DRV and ATV therapy, respectively [45,46]. Mutations at protease residue 82 are observed in patients treated with RTV and SQV, and the G48V mutation results in resistance to SQV and ATV [47,48]. The I84V mutation is one of the severe primary resistance mutations causing cross-resistance to most PIs [49]. Thus, a range of primary resistance mutations are selected, some of which are unique to a single PI, whereas others confer resistance to two or more PIs.

Mutations in HIV-1 protease, either within or outside the active site, can decrease the binding affinity of inhibitor molecules in a complex, interdependent and cooperative manner. When a protease variant binds to an inhibitor, the structure of the protease adjusts to accommodate the inhibitor by rearranging the interactions not only at the mutated residue but also throughout the protein [50–52].

Analysis of protease inhibitor complexes has shown that the structure of HIV protease is highly plastic [4,35,51]. The conformational change observed in the mutant protease is not always just around the vicinity of the mutation. Various conformations found in crystal structures are probably the combined effect of the nature of the inhibitor and the combination of mutations present in the protease. Whether there is a major conformational change in the protease backbone or not, the drug resistant mutation(s) does have an impact on the binding affinity to the inhibitor.

The rearrangement of the backbone can be observed either in the entire protease or in some parts of the protease, as in flap region or P1 loop region, or just locally around the mutated residue [53]. Previous studies involving the drug resistant inactive variant of protease (D25N and V82A) with the inhibitors SQV and RTV showed that the binding of the inhibitor is compromised because of the drug resistant mutation, V82A [51]. In addition to the direct loss of van der Waals contacts between the inhibitors and the protease as a result of the V82A mutation, the mutant protease also undergoes conformational changes as observed by the large shifts in the Cα backbone compared to the wild-type structure. In another study [54], the binding of inhibitors, APV, DRV, ATV and SQV to the protease variant containing L10I, G48V, I54V and V82A mutations has shown large changes in the flap regions of the protease. In this case, the changes in the flap region are attributed to the two mutations present in the flap (G48V and I54V), which may have locked the conformation of the flaps. The study by Munshi *et al*. [50] revealed that the 80’s loop is intrinsically flexible and that mutations in this loop are not necessary to result in conformational changes. Conformation of the P1 loops in the inhibitor-protease complex depends mainly upon the nature of the bound inhibitor and may be influenced by mutations in the protease [50]. This means that the rearrangement of the protease also depends on the relative shifts and tilts in the bound inhibitor. For instance, in the study [39] involving the V82T/I84V protease variant bound to APV and DRV, minor changes in the backbone of the protease were observed compared to the wild type. The P1 loop of only one monomer is shifted in the mutant structure corresponding to the shift and tilt of DRV whereas, P1 loops of both the monomers of the protease are shifted in the APV mutant protease structure corresponding to the shift and tilt of APV. Additionally, there are minor backbone rearrangements in the crystal structures of the V82T/I84V protease variant with ATV and SQV [54] where the shifts and tilts of the inhibitors account for the altered interactions and hence, to the reduced affinity of the inhibitor. In a study by Konvalinka *et al*. [55], the impaired binding of the inhibitor to the drug resistant protease is explained by a change in hydrogen bonding pattern due to a substantial shift of the aminophenyl moiety of DRV.

### 3.3. Contribution of Protease Mutations outside the Active Site

Structural analyses of inhibitor complexes have been useful in the elucidation of the mechanism by which active site mutations confer resistance to PIs [37,38,51,56]. Notably, the substrate envelope hypothesis has helped explain the change in molecular recognition in resistant protease, where the enzyme evolves to resist inhibitor binding but continues to recognize and bind its natural substrates [37]. However, the protease mutates extensively in the regions beyond active site, and these non-active site mutations have been known to greatly contribute to drug resistance. The mechanism by which the mutations outside the active site confer resistance remains elusive. Some of these mutations are primary drug resistant mutations and others have been suggested to contribute to drug resistance when present along with other major mutations.

Of the 99 positions in each monomer, nearly 37 are known to be invariant (with mutation frequencies <0.5%) and 17 positions are sites of non-treatment related polymorphisms [41,42,57]. Nearly 45 positions in each monomer have been implicated in drug resistance. Of these 45 positions, mutations at 26 positions have been shown to significantly decrease susceptibility to one or more PIs and the others are polymorphic mutations that occur more frequently when associated with inhibitor therapy [42,58]. Furthermore, almost 60% of these 26 positions fall outside the active site region (Table 2). Thus, excluding the invariant positions and including the polymorphic sites associated with drug resistance, almost 40–45% of the protease sequence is implicated in contributing to drug resistance [41,42,57], and a staggering 60–63% of the sequence has been known to vary in patient isolates.

Various groups, in the past, have studied thermodynamic, structural and kinetic parameters of various combinations of the major drug resistant mutations and contributory or associated non-active site secondary mutations in recombinant protease system [59–64]. Almost all these studies have shown that the effect of major drug resistance mutations is highly diminished in the absence of paired secondary non-active site mutations. Although the mechanism by which these diversely placed non-active site residues orchestrate altered inhibitor-binding remains largely unknown, some residue-specific explanations and suggestions have been put forth [65,66]. One of the reasons for this altered binding has been suggested to lie in the internal dynamics and inherent plasticity of HIV-1 protease [60,67,68]. Some of these mutations may induce conformational perturbations in the enzyme, altering binding of the inhibitors. Kinetic studies conducted on various permutations and combinations of active and non-active site protease mutants have shown that many of these protease variants have decreased catalytic efficiencies, resulting from either increased *K_M_* values or reduced turnover rates or a combination of both [60,69]. Some mutations, e.g., L90M, have been shown to make protease a better enzyme for one substrate over the other in a clade specific manner [52,70].

### 3.4. Impact of the Co-evolution of Protease Cleavage Sites on Resistance

Following accumulation of resistance mutations within the protease, mutations also develop within the substrate cleavage sites in Gag and Gag-Pro-Pol [71,72]. Mutations were first reported within the NC-p1 and p1-p6 cleavage sites [71,73,74]. Additionally, associations between specific mutations in the protease and the cleavage sites have been reported previously, and were demonstrated to alter susceptibility to various PIs [71,73–76]. The A431V mutation within the NC-p1 cleavage site and L449F in the p1–p6 cleavage site selected during the evolution of PI resistance were observed to correlate with V82A and I50V protease resistance mutations, respectively [71,76].

Gag processing is enhanced by the A431V and I437V mutations within the NC-p1 cleavage site [77,78]. In fact, there were clear structural changes that increased binding of the A431V NC-p1 site with the V82A protease [79]. Recently though, both A431V and I437V have been shown to directly increase resistance, possibly as a result of this enhanced Gag processing [78,80]. Similarly, the L449F mutation within the p1–p6 cleavage site has been shown to increase processing at this cleavage site [76,77,81]. Likely, the change from a smaller amino acid to a larger Phe improves van der Waals contacts contributing improved Gag processing. These studies revealed that the p1–p6 cleavage site mutations are associated with the NFV-resistant D30N/N88D protease mutations. In addition to these, several other correlations between the NC-p1 and p1–p6 cleavage site mutations and primary drug resistant mutations were observed [82]. These cleavage site mutations have been demonstrated to be compensatory in nature by improving replicative capacity and/or Gag processing [77,79]. Other cleavage site mutations, including I437V and P453R, have now been well documented and are associated with several major protease resistance mutations [76,82,83]. This suggests a mechanism whereby decreased interactions between cleavage sites and mutant protease can be offset by compensatory mutations within the cleavage sites leading to improved binding and processing. This implies that with prolonged PI therapy, evolution of protease cleavage sites could be a fairly frequent mechanism for maintaining viral fitness even as the virus evolves resistance to PIs.

Studies have shown that co-evolution of substrate cleavage sites and protease mutations also contribute to PI resistance [78,82]. Primary PI resistance mutations, especially in the active site, reduce both protease catalytic efficiency and viral replicative capacity (RC) [84–87]. Several studies have demonstrated that the evolution of compensatory mutations within cleavage sites leads to improved viral fitness compensating for the loss in fitness resulting from the protease resistance mutations [71,72,74,88]. However, significant differences were not observed in viral fitness with protease resistance mutations in the presence and absence of mutations within the Gag cleavage sites [82]. More recently, Larrouy *et al*. observed that baseline cleavage site mutations, in treatment-naïve patients, were significantly linked to virological outcomes [89]. More specifically, mutations at Gag 128 within the MA-CA and Gag 449 within the p1–p6*^gag^* cleavage sites were associated with low virological response whereas mutations at Gag-Pol 437 within the TFP-p6*^pol^* were frequent in patients achieving virological response [89]. In a recent study, Parry *et al*. demonstrated that mutations in the matrix and partial capsid in the N-terminal regions of Gag fully restore RC to WT levels and thus play a key role in fitness [90]. However, these mutations significantly enhanced resistance to PIs even in the absence of PI resistance mutations in the protease [90]. Thus, the evolution of mutations within the cleavage sites and outside play an important role in the development of resistance and affect virological response during therapy.

Statistical analysis on the effect of the observed correlations on phenotypic susceptibilities to various PIs showed that these correlations were observed to significantly affect PI susceptibilities. In most instances, a significant decrease in phenotypic susceptibility to particular PIs was observed. Although mutations at either Gag 431 or Gag 437 were not associated with D30N/N88D protease mutations, significantly lower PI susceptibilities were observed. A similar trend was also observed with Gag A431V and the L90M protease mutation. Mutations at either of these residues within the NC-p1 cleavage site likely directly enhance resistance to PIs, as was observed and demonstrated previously [78,91]. At least in the case of the Gag A431V mutation, this is likely due to enhanced Gag processing at this site as demonstrated by Nijhuis *et al*. [80]. Thus, Gag cleavage site mutations enhance resistance to PIs in combination with primary drug resistance mutations in the protease. A detailed review of the role Gag cleavage sites on protease inhibitor resistance by Clavel and Mammano is included in this issue [92].

## 4. Altered Pathways to Drug Resistance between the HIV-1 Clades

**Figure 4.**
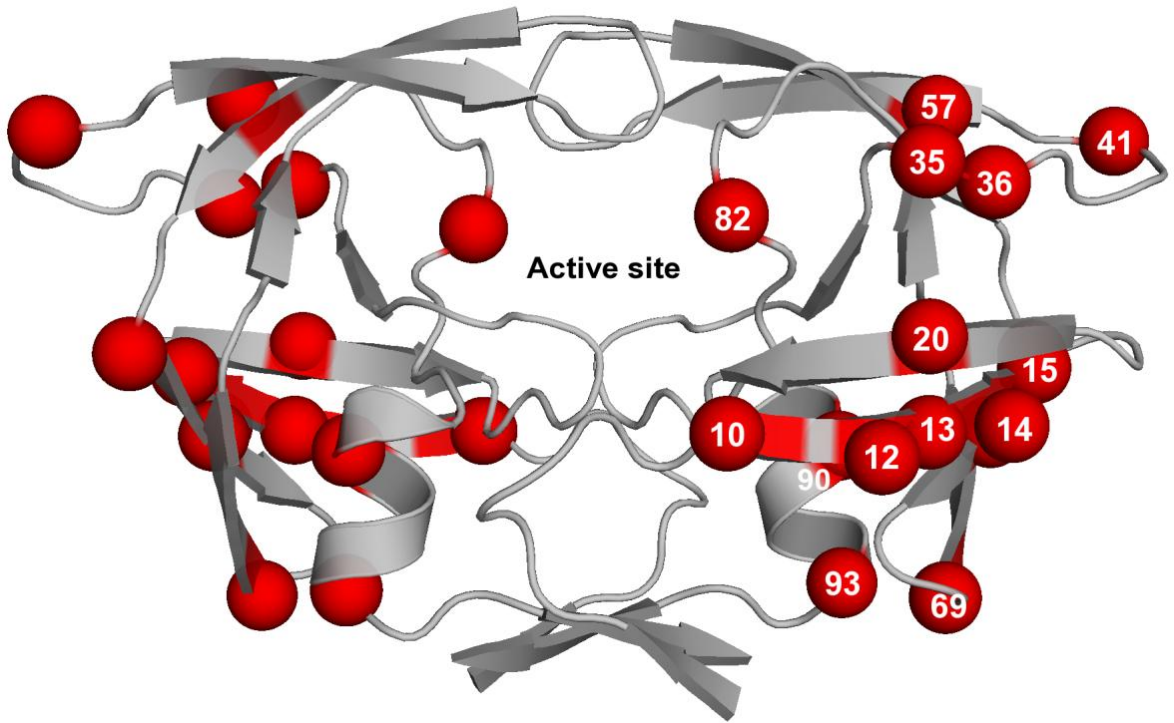
HIV-1 protease is a homodimer with the catalytic active site formed at the dimeric interface. The majority of residues that differ between various HIV-1 clades map to positions that are outside the active site. Red spheres represent amino acid positions and are indicated only on one monomer for clarity.

Based on genomic diversity, HIV-1 has been classified into nine clades (A, B, C, D, F, G, H, J, and K) and 43 circulating recombinant forms (CRFs) [93,94]. The protease amino acid sequences between clades vary up to about 10%. A number of these amino acid variations have been associated with PI resistance in clade B (Table 3). With the exception of clade G, which has an active site amino acid substitution when compared to clade B, all sequence variations within other clades map to positions outside the active site (Figure 4). While currently available PIs are effective against different HIV-1 clades very few studies have been carried out to understand the effect of clade specific sequence variations on the emergence of drug resistance.

Despite the lack of data on pathways to resistance on non-B clade proteases, a number of studies focusing on sequence polymorphisms in protease have highlighted differences in biochemical and structural profiles as well as viral replication in non-B clade viruses when compared to clade B. Enzyme kinetics studies show higher *K_M_* values, 1.4-fold, for clade A and lower *K_M_* values, 2.6-fold and 3.4-fold, for clade C and G protease when compared to clade B and indicates that affinity for substrates might be different between clades [95]. Studies carried out on CRF01_AE have shown that while *K_M_* values were comparable to that of clade B the catalytic turnover rates (*k_cat_)* were significantly lower in CRF01_AE protease [96]. Crystal structures of the AE protease indicate that the flap hinge region of the protease is less flexible when compared to clade B protease that might lead to the lower turnover rates observed in the AE protease. Thus, currently available data suggest that despite the fact that sequence variations in non-B proteases map outside the active site, they play a role in modulating enzymatic activity.

*In vitro* studies carried out by Holguín and colleagues have shown that M36I, a polymorphism found in most non-B clade proteases, increased viral replicative capacity in the absence of drug pressure while both K20I and M36I increased viral replication under drug pressure [97]. This suggests that the replicative advantage resulting from sequence polymorphisms could allow non-B clade variants to spread even under drug pressure.

Binding studies carried out on clade A, C and G by Velazquez-Campoy and colleagues [95] and on CRF01_AE by Bandaranayake and colleagues [96] show that the wild type non-B clade proteases have an inherent weaker affinity for a number of currently available FDA approved PIs. Though these observations are indicative that background polymorphisms observed in non-B clade protease can affect inhibitor binding, clinical data suggest that currently available PIs can be just as effective against non-B clade variants as they are against clade B. However, the weaker affinity for inhibitors observed may make resistance easier to occur for non-B clade viruses against the current regime of PIs. This idea has been further strengthened by the observation of altered PI resistance pathways in some non-B clade proteases. Two distinct examples of altered resistance pathways in non-B clade variants have been in clade C, which develops L90M, and in CRF01_AE, which develops N88S, in response to NFV therapy whereas clade B develops D30N, N88D [98,99]. Work carried out on CRF01_AE suggests that the protease has an inherent weaker affinity for NFV and thus, the reduced affinity for NFV might allow the CRF01_AE protease to confer resistance through N88S, non-active site mutation, whereas clade B protease which has a higher affinity for NFV requires a combination of an active site and non active site mutations, D30N and N88D, in order to effectively disrupt NFV binding.

While currently available PIs are highly effective in treating all clades, different clades might vary in how they respond to PI therapy. Resistance to PIs remains to be a major challenge in the effective treatment of HIV-1 and becomes even more relevant in geographic locations where administering optimal treatment regimens is difficult. Given that non-B clade HIV-1 variants are more prevalent across the world continued studies on non-B clade proteases are important to elucidate how sequence variations influence protease activity and the emergence of resistance mutations. Such studies would add to our current understanding of drug resistance and help formulate effective global treatment strategies.

## 5. The Atomic Energetics of Drug Resistance

At the roots of the molecular basis for drug resistance are the alterations in the atomic interactions between the PI and the resistant variant of HIV-1 protease. Free energy calculation and decomposition techniques are providing new insights into protein-ligand interactions [100–106]. Specifically, the MM-PB/GBSA method [107,108] has been applied in several cases to study the molecular mechanism of HIV-1 protease drug resistance [109–112]. Compared to the classic free energy perturbation and thermodynamic integration methods [100,102,113,114], MM-PB/GBSA is computationally less demanding and a more practical solution for scanning the chemical compound library to discover lead compounds for potential new inhibitors [115]. The MM-PB/GBSA method combines molecular mechanism energies and solvation energies to estimate the absolute protein-ligand binding energy, allowing for the elucidation of which interactions contribute the most to the binding energy. Most of the interactions are calculated by the atom pairs allowing decomposition of the interaction energy to the residues of the protease or the functional groups of inhibitors [116,117]. Such decomposition helps to elucidate the protease drug resistance mechanism on an atomic level and generates valuable suggestions on modification of the current inhibitors for improvement.

Wang *et al*. [111] calculated the binding energy between the wild-type protease and the inhibitors APV, SQV, RTV, IDV, NFV and a substrate of eight amino acid residues. By comparing energy profiles and the differences at each protease residue, it was suggested that the drug resistant mutations are more likely to occur at protease residues that interact more favorably with inhibitors than the substrate. They proposed that a strategy for new inhibitor design is to develop compounds that interact most favorably with the well conserved protease residues. By considering a residue’s energy contribution to the binding and the site’s sequence variability, Wang *et al*. defined an empirical parameter to identify the drug resistant mutations. In a study of protease binding with seven cyclic ureas [118], Mardis *et al*. reproduced the U-shaped trend of binding free energy as a function of aliphatic chain length of the inhibitors. Their results also demonstrated that in treating the desolvated system such as the protein binding site, the finite difference Poisson-Boltzmann model [119] are more accurate than the generalized Born method. Recently, Hou *et al*. calculated the binding affinities between APV, TMC-126, DRV (with the WT protease and a multi-drug resistant variant (V82F/I84V) [110]. Stoica *et al*. calculated the binding affinities between SQV with wild-type protease and three different drug resistant variants (G48V, L90M, G48V/L90M) [109]. Cai *et al*. calculated the binding affinities between DRV with wild-type protease and two multi-drug resistant variants (L10I/G48V/I54V/V82A, V82T/I84V) [112]. The largest uncertainty came from the evaluation of the vibrational entropy. Hou *et al*. [110] showed that by excluding the entropy terms, the predicted binding free energies were in better correlation with the experimental energies. In these applications of the MM-PB/GBSA methods to the energetic features of protease binding with inhibitors, the predicted absolute binding free energy were in good agreement with the experimental results. They predicted the ranking of the binding affinities correctly. The more rigorous thermodynamic integrations method showed better prediction on the relative binding energy [112].

Overall, by free energy decomposition analysis, the drug-resistant mutations were found to distort the geometry of the binding site and hence weakened the binding affinity of the inhibitors [110,112]. Van der Waals interaction has been found to have the biggest contribution to the protease-inhibitor binding affinity [109–112]. Modification of current inhibitors to design more robust inhibitors can be attained by evaluating changes in van der Waals interaction energy between the protease and each atom of the inhibitors [112]. The electrostatic energy becomes less important than the van der Waals because a more favorable coulombic interaction was usually associated with a higher penalty for the solvation energy [109–112]. Charge optimization studies have been carried out to find the best balance between the coulombic interaction energy and the polar solvation energy to generate compounds with highest electrostatic interactions energy with the protease [120–122].

## 6. Incorporating the Substrate Envelope Constraint in Structure Based Drug Design

Developing robust HIV-1 PIs that avoid drug resistance has proven a challenging task, and the substrate envelope hypothesis provides an approach to solving this problem. A survey of five approved drugs using quantitative measures of the bound inhibitor outside the substrate envelope indicated that the exterior volume of the inhibitors correlated with the loss of affinity to mutant proteases [123]. A recent study of the inhibitor R01 suggested that individual mutations did not confer drug resistance, but when multiple sites protrude beyond the envelope collectively, resistance may occur [124]. The drug DRV, which is structurally similar to APV, demonstrated improved potency with the resistant mutants which is attributed to both DRV’s high binding affinity and that DRV lies within the substrate envelope [39].

The ability of the substrate envelope to correlate with resistance mutations prompted the use of substrate envelope constraints in the design of new inhibitors [24,29,35,125,126]. Inhibitors were designed by varying different groups on the hydroxyethylamine scaffold using three different methodologies: Two computational methods incorporated structural constraints of the substrate envelope as an *a priori* consideration during the design stage of the inhibitors while the third method, structure activity relationship (SAR), did not include the substrate envelope constraint explicitly in its design. The first computational design [126] based on optimized docking resulted in two good candidates exhibiting flat affinity profiles against multi-drug resistant mutants. But these inhibitors have binding affinity in the nM range. The second computational study systematically explored the combinatorial space for three constituent R groups on the hydroxyethylamine scaffold [29] in two rounds of inverse drug design, synthesis, testing, and retrospective structural analysis. The first round produced compounds with *K*_i_ in the range of 26 μM–30 nM, which was improved to *K*_i_ of 4.1 nM–14 pM in the second round compounds. Majority of these inhibitors, whether they are nanomolar or picomolar inhibitors, have flatter resistance profiles against drug resistant variants. Although the inhibitors designed using SAR approach [125] resulted in inhibitors with picomolar affinity to the wild-type protease they all lose significant affinity while binding to the drug resistant protease variants. These studies validated the use of the substrate envelope hypothesis [35] for the development of therapeutics with low susceptibility to resistance mutations in HIV-1 protease and have yielded several leads for potential new drugs.

Application of the substrate envelope hypothesis to development of therapeutics to other quickly evolving drug targets is beginning to emerge. In a recent study [127], the hypothesis has been applied to five prospective drug targets from a diverse set of diseases, and the volume of inhibitors protruding beyond the native substrate specified envelope correlates with average mutation sensitivity. This suggests that inhibitor design for these enzymes would benefit from a similar reverse engineering strategy as was implemented in the case of HIV-1 protease. The substrate envelope model has also been applied in the development of tenofovir, a reverse transcriptase inhibitor [128]. Similar to the case of HIV-1 protease, the drugs AZT and 3TC protrude beyond the consensus volume, creating an opportunity for the reverse transcriptase to develop resistant mutations. The newer drug, lacking such protrusions, is expected to evade resistance mutations as an improvement over its predecessors. Thus the substrate envelope hypothesis appears to be a valid general strategy for avoiding drug resistance.

## 7. Conclusions

Drug resistance in HIV protease is a subtle change in the balance of recognition events between the relative affinity of the HIV protease to bind inhibitors and its ability to bind and cleave substrates. Viral maturation involves the cleavage of Gag and Gag-Pro-Pol polyproteins by the viral protease in a complex, interdependent, and order-specific series of recognition and processing events. Mutations that confer resistance while balancing viral fitness have long been identified, both within and outside the active site of the enzyme, although their direct mechanism of action is not always well understood. Most changes confer resistance not only by altering a direct contact with a protease inhibitor, but also by conferring subtle changes in the structure and energetics throughout the active site. As many mutations occur simultaneously in complex combinations within a single protease variant, they are most likely altering both the structure and dynamics of this enzyme. Recent data also implicate that mutations at the protease cleavage sites as well as remote sites within Gag contribute to HIV protease drug resistance, possibly without altering viral fitness. The mechanism by which these changes confer resistance is likely an alteration in the balance of recognition events of the entire viral system and how the virus interacts within the host cell. Subtle changes between viral clades also alter this balance. Taken together, all these changes necessitate taking a comprehensive systems approach to understanding the molecular basis for drug resistance in the highly interdependent molecular system of HIV.

HIV-1 protease, with its ability to recognize and cleave diverse substrate sequences, has proved to be a resilient drug target. If targeted optimally in a manner that is evolutionarily constrained, the protease may be less susceptible to resistance. The substrate envelope hypothesis described a structure based drug design approach that decreases the probability of drug resistance by understanding the functional complexes of the HIV protease bound to its cleavage sites. The substrate envelope was then used as an added constraint in optimizing existing inhibitor scaffolds and designing novel robust inhibitors. Other strategies, such as focusing on main chain interactions, also may lead to similar results. A robust inhibitor is one that successfully inhibits a resilient target and does not quickly lose effectiveness due to resistance. Such an inhibitor may bind only to critical regions within the target that would be essential for function and thus intolerant to change. Of the currently prescribed PIs, DRV is the closest to being such a robust inhibitor. However, with the continuous evolution of HIV strains, development of other potent and robust HIV-1 protease inhibitors is highly warranted. In addition to drug resistance, other factors such as bioavailability, *in vivo* stability, and toxicity must also be taken into consideration when selecting a drug candidate for development.

## Figures and Tables

**Table 1.** Binding affinity [29,35] and antiviral potency [36] of FDA approved HIV-1 protease inhibitors.

	*K*i (nM)				
Inhibitor	WT/Q7K	L10I, G48V, I54V, L63P, V82A	D30N, L63P, N88D	L10I, L63P, A71V, G73S, I84V, L90M	Antiviral EC_50_ (nM)
Saquinavir	0.065	90	1.0	78	26
Indinavir	0.18	34	0.73	21	40
Ritonavir	0.055	3.0	0.46	2.8	65
Nelfinavir	0.28	15	3.5	19	71
Amprenavir	0.10	0.15	0.21	1.40	44
Lopinavir	0.005	6.1	0.04	0.90	10
Atazanavir	0.046	0.33	0.009	0.49	15
Tipranavir	0.088	0.014	0.001	0.032	500
Darunavir	0.008	0.005	0.041	0.025	1

**Table 2.** The major non-active site mutation positions which cause decreased susceptibility to one or more PIs [22]. The known polymorphisms are listed for subtype B [40].

**Positions**	**Wild-type Amino Acid**	**Most Frequent Mutations**	**Polymorphic/Non-polymorphic**
10	L	FI	(L10I) Polymorphic
			(L10F) Non-polymorphic
11	V	L	Non-polymorphic
20	K	T	Non-polymorphic
33	L	F	Non-polymorphic
35	E	GN	Non-polymorphic
43	K	T	Non-polymorphic
46	M	IL	Non-polymorphic
54	I	ALMSTV	Non-polymorphic
58	Q	E	Non-polymorphic
73	G	CST	Non-polymorphic
74	T	PS	Non-polymorphic
76	L	V	Non-polymorphic
88	N	DS	Non-polymorphic
89	L	V	Non-polymorphic
90	L	M	Non-polymorphic

**Table 3.** Protease positions that differ between HIV-1 clades. The line highlighted in orange shows amino acid substitutions that are associated with inhibitor resistance in clade B.

**Position**	**10**	**12**	**13**	**14**	**15**	**20**	**35**	**36**	**41**	**57**	**61**	**69**	**82**	**89**	**93**
**Clade B**	L	T	I	K	I	K	E	M	R	R	Q	H	V	L	I
**Resistance Associated Mutations in clade B**	I		V			I/R	D	I					A	M	L
**Clade A1/A2**	I		V	R			D	I	K	K		K		M	
**Clade C**		S			V			I/V	K/N			K		M	L
**Clade D**	V		V					I	K						
**Clade F1/F2**	V/I	S			V	R	D	I/V	K	K	N			M	
**Clade G**	I		V	R		I	D	I	K			K	I	M	
**Clade H**			V			R		I	K					I	
**Clade J**			V	R		R		I	K		E			M	
**Clade K**	I				V	R		I	K					M	
**CRF01_AE**			V			R	D	I	K			K		M	
**CRF02_AG**	V/I		V	R		I		I	K			K		M	
